# Nineteen percent of meniscus repairs are being revised and failures frequently occur after the second postoperative year: a systematic review and meta-analysis with a minimum follow-up of 5 years

**DOI:** 10.1007/s00167-021-06770-x

**Published:** 2021-10-20

**Authors:** Conradin Schweizer, Carola Hanreich, Philippe M. Tscholl, Robin Ristl, Sebastian Apprich, Reinhard Windhager, Wenzel Waldstein

**Affiliations:** 1 Department of Orthopaedic and Trauma Surgery, Vienna General Hospital, Medical University of Vienna, Vienna, Austria; 2 Section for Medical Statistics, Center for Medical Statistics, Informatics and Intelligent Systems, Medical University of Vienna, Vienna, Austria; 3 Division of Orthopedics and Trauma Surgery, Geneva University Hospitals, ReFORM (Reseau Francophone Olympique de la Recherche en Médecine du Sport), IOC Research Centre for Prevention of Injury and Protection of Athlete Health, Geneva, Switzerland

**Keywords:** Meniscus repair, Meniscus failure, Meniscus repair technique, Anterior cruciate ligament reconstruction

## Abstract

**Purpose:**

Meniscus repair has gained increasing interest over the last two decades as loss of meniscus tissue predisposes to early onset knee arthritis. Although there are many reports of meniscus repair outcome in short-term studies, data on the long-term outcome of meniscus repair are still scarce. The purpose of this meta-analysis was to evaluate the overall failure rate of meniscus repair with a minimum follow-up of 5 years. Additionally, possible factors influencing meniscus repair outcome were assessed.

**Methods:**

PubMed and Scopus were searched for studies of the last 20 years reporting on meniscus repair outcome with a minimum follow-up of 5 years. The study was performed following the Preferred Reporting Items for Systematic Reviews and Meta-Analyses guidelines. The search terms used for this study were ([meniscus OR meniscal] AND repair). Titles and abstracts were evaluated by two authors independently. Using meta package of R (version 3.6.2), random-effect models were performed to pool failure rates. Subgroup analyses were performed and effect estimates in form of an odds ratio with 95% CIs were established.

**Results:**

In total, 12 studies with 864 patients were included. Degenerative tears were excluded in two studies and one study only included traumatic meniscus tears. Other studies did not state whether the cause of meniscus tear was degenerative or traumatic. Studies reporting meniscus repair outcome on root repairs, revision anterior cruciate ligament reconstruction, discoid menisci or ramp lesions were excluded. Revision surgery was used as failure definition in all included studies. The overall failure rate of meniscal repair at a mean follow-up of 86 months was 19.1%. There was no significant difference in meniscus repair outcome when performed in combination with anterior cruciate ligament reconstruction compared to isolated meniscus repair (18.7% vs. 28%; n.s.) or when performed on the lateral meniscus compared to the medial meniscus (19.5% vs. 24.4%; n.s.). There was no significant difference of meniscus repair outcome between vertical/longitudinal tears and bucket-handle tears (n.s.). Thirty-six percent of meniscus repair failures occur after the second postoperative year. The only significant finding was that inside-out repair results in a lower failure rate compared to all-inside repair (5.6% vs. 22.3%; *p* = 0.009) at 5 years.

**Conclusion:**

The overall meniscus repair failure rate remains nineteen percent in long-term studies. The cause of failure is poorly documented, and it remains unclear whether failure of the meniscus repair itself or additional adjacent tears lead to revision surgery. Despite the given technical advantages of all-inside repair devices, this meta-analysis cannot demonstrate superior outcomes compared to inside-out or outside-in repair at 5 years.

**Level of evidence:**

IV.

## Introduction

Meniscus surgeries are among the most frequently performed interventions in orthopedic surgery [8]. The importance of the menisci for a physiologic function of the knee joint is well understood [18, 28, 37, 42]. Furthermore, there is well-established evidence that loss of meniscus tissue predisposes to early onset knee arthritis [4, 17, 23, 35, 39]. Thus, meniscus repair has gained great interest over the last two decades leading to a significant increase of meniscus repair compared to meniscectomy [2].

Meniscus tears may occur due to a relevant trauma or may develop over time as part of a degenerative process. Traumatic meniscus tears often occur in combination with other injuries to the knee such as rupture of the anterior cruciate ligament (ACL). In an attempt to restore physiologic knee function in younger patients these acute meniscus injuries are often sutured. The optimal treatment of degenerative meniscus tears has been a matter of intense research over the last decades. There is consensus that in absence of recurrent knee catching or blocking, surgical treatment should not be considered the first-line intervention for degenerative meniscus tears [1]. Most of the time when surgery is considered, partial meniscectomy remains the only treatment option.

In comparison to open meniscus repair, arthroscopic meniscus repair has many favorable effects such as minimal trauma, short operation time and early recovery, respectively [10]. Numerous arthroscopic meniscus repair devices have been developed in an attempt to facilitate surgical procedures and improve the outcome of meniscus repair. While the majority of studies reported short-term results of meniscus repair, only a few studies have described the long-term outcome [16]. A previous meta-analysis reported that approximately 30% of all meniscus repair failures occur after the second postoperative year [21]. Lee et al. even showed a deteriorating success rate after 2 years postoperatively [14].

Due to the rising popularity of meniscus repair, improvement of surgical techniques and little available literature on long-term results, an analysis of possible predictors on meniscal repair outcome is of high interest. A better understanding of the expected outcome will facilitate a more differentiated approach on the optimal surgical treatment. The aim of this study is to describe the overall failure rate of meniscus repair with a minimum follow-up of 5 years. Furthermore, the associations of concomitant anterior cruciate ligament repair, laterality, repair technique, tear configuration, patients’ age and rehabilitation protocols on meniscus repair outcome are determined. Additionally, the time of meniscus repair failure in long-term studies is described. This study is the most comprehensive and detailed analysis of meniscus repair outcome at a minimum follow-up of 5 years. The results of newer generation all-inside repair devices and different tear configurations are analyzed.

## Material and methods

### Literature search

In accordance with the guidelines of the preferred reporting items for systematic reviews and meta-analyses (PRISMA) statement [20], a systematic literature review using both PubMed and Scopus on studies published between January 2000 and October 2020 was performed. The search terms used for this study were ([meniscus OR meniscal] AND repair).

### Study eligibility

Studies were included if they met the following criteria: (1) minimum follow-up of 5 years, (2) report on meniscus repair failure, (3) a cohort size of greater than ten patients and (4) a consecutive follow-up rate of more than 70%.

Biomechanical and cadaveric studies, technical notes, letters to the editor, review articles, meta-analyses and case reports were excluded. Studies with an average age younger than 18 years or published in languages other than English were also excluded. Failures were defined according to the definition of every individual study. Studies reporting meniscus repair failure with root repairs, revision anterior cruciate ligament reconstruction (ACLR), discoid menisci or ramp lesions were excluded.

### Study selection and quality assessment

Two authors (CS and CH) evaluated all titles and abstracts of the retrieved studies independently. Any disagreement between the two authors were resolved by mutual agreement. The methodological index for non-randomized studies (MINORS) [30] was used to assess the quality of all included studies (Table 1).

**Table 1:** The methodological index for non-randomized studies (MINORS) of all included studies (*n* = 12)

Methodological index for non-randomized studies (MINORS)														
Year	Authors	1	2	3	4	5	6	7	8	9	10	11	12	Total
2019	Billières J et al. [5]	2	1	2	2	1	2	1	0	–	–	–	–	11
2014	Bogunovic L et al. [6]	2	1	2	2	1	2	1	0	–	–	–	–	11
2005	Lee GP et al. [14]	2	2	2	2	1	2	1	0	–	–	–	–	12
2009	Logan M et al. [15]	1	1	2	1	1	2	2	0	–	–	–	–	10
2006	Majewski M et al. [19]	2	2	2	2	1	2	1	0	–	–	–	–	12
2015	Pujol N et al. [25]	2	2	2	2	1	2	1	0	–	–	–	–	12
2000	Rockborn P et al. [26]	2	2	2	2	1	2	1	0	2	2	2	2	20
2007	Siebold R et al. [29]	2	2	2	2	1	2	1	0	–	–	–	–	12
2016	Solheim E et al. [31]	2	2	2	2	2	1	2	0	–	–	–	–	13
2015	Steadman JR et al. [33]	2	2	2	2	1	2	1	0	1	2	1	2	18
2004	Steenbrugge F et al. [34]	2	1	2	1	1	2	1	0	1	0	0	1	12
2014	Westermann RW et al. [41]	2	2	2	2	1	2	1	0	–	–	–	–	12

### Data extraction

A predefined data extraction sheet was used to extract relevant information on meniscus repair failures, patient demographics, tear configurations, repair techniques and devices, the ACL status and rehabilitation protocols, respectively. When more than one follow-up assessment was available, the latest follow-up was included. To avoid the overlap of cohorts, studies with the same surgeon were excluded if time of surgery overlapped with others. In such cases, studies with larger cohorts were selected. In one study, only a meniscus repair subgroups was analyzed, to avoid overlapping cohorts [6]. If necessary, attempts were made to contact the authors to either receive missing data or clarify open questions. Revision surgery was used in all included studies for the definition of meniscus repair failure.

### Statistical analysis

Random-effect models (REM) using the Restricted Maximum Likelihood Method (REML) as *τ*^2^ were performed to pool failure rates (≙incidence rate (IR) as effect size) and to establish 95% confidence intervals (CI) [13]. When considered relevant and more than three studies per group were available, REM subgroup analyses were performed. To assess the influence of laterality on the failure rate, effect estimates in form of an odds ratio (OR) with 95% CIs by means of a binary REM were established. Only studies with cohort sizes of *n* ≥ 5 in subgroups were included for calculating OR. Continuity correction of 0.5 in studies with zero cell frequencies was applied. Hartung Knapp adjustment for REM was used in all analyses [22]. Heterogeneity was estimated using *I*^2^ statistics for all analyses [9]. *p* values ≤ 0.05 were considered statistically significant. The meta package of R (version 3.6.2) was used for all analyses.

## Results

**Fig. 1:**
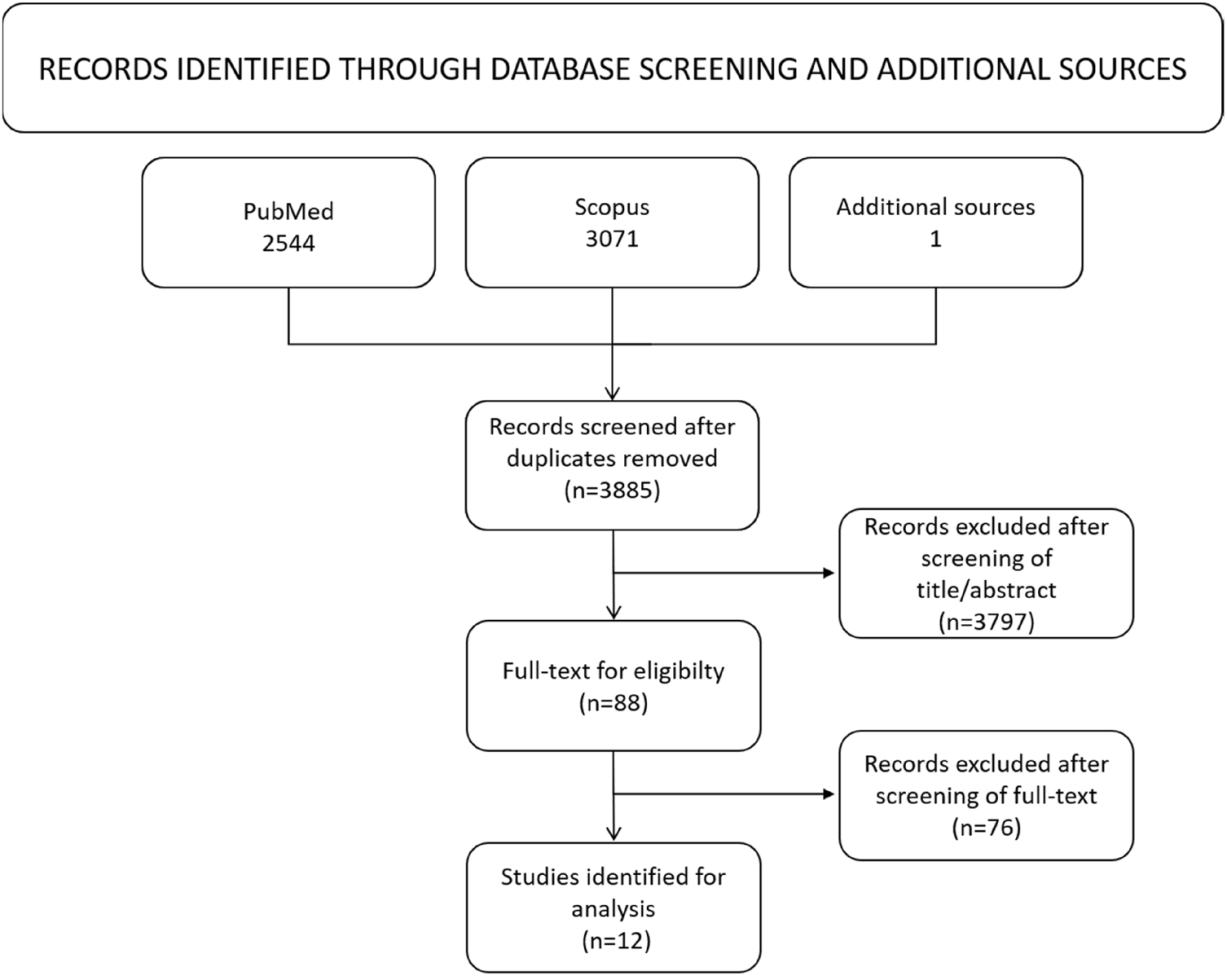
Flow chart according to the PRISMA guidelines

The initial literature search identified 3885 studies. After applying the inclusion criteria of this study, a total of 12 studies was included (Fig. 1). The level of evidence was III in five and IV in seven studies, respectively. The duration of minimum follow-up ranged from 60 to 144 months (mean 86 months). Study details are shown in Table 2 and tear/suture characteristics in Table 3, respectively. All included studies used revision surgery as failure definition. One study [14] additionally included clinical symptoms and one study [25] additionally used arthro-CTs for the definition of meniscus repair failure. Two study [15, 19] excluded menisci with signs of tissue degeneration whilst another study [31] only included traumatic meniscus injuries. The remaining studies did not mention whether the cause of meniscus repair was degenerative or traumatic**.** Meniscus repair outcome was analyzed in 864 patients, yielding an overall failure rate of 19.1% (165/864). Of available data, 32% female and 68% male patients were included.

**Table 2:** Patient demographics, study details and failure assessment extracted from all studies (*n* = 12)

Year	Author	Journal	Subgroups	Ø Age^a^	Cohort size (*n*)	Female:Male	Ø Follow-up (months)	Range	Repair technique	Simultanous ACLR (n/% of entire cohort)	Repair Device	Failure assessment	Failure rate (%)
2019	Billières J et al. [5]	KSSTA	No subgoup	28.4^b^	13	2:12^a^	102^b^	84–144	Open repair	0	No all-inside	Revision surgery	15.0
2014	Bogunovic L et al. [6]	JBJSA	Isolated MR	26.8	26	10:16	84	60–150	All-inside	0	FasT-Fix™ (Smith & Nephew)	Revision surgery	12.0
2005	Lee GP et al. [14]	AJSM	No subgoup	Not specified	28	Not specified	79	65–88	All-inside	28 (100%)	Meniscus Arrow (Bionx Implants)	Clinical or revision surgery	29.0
2009	Logan M et al. [15]	AJSM	No subgoup	23.2	42	13:29	108	65–151	Inside-out	Not specified	No all-inside	Revision surgery	24.0
2006	Majewski M et al. [19]	AJSM	No subgoup	29.8	88	34:54	120	60–204	Outside-in	0	No all-inside	Revision surgery	24.0
2015	Pujol N et al. [25]	KSSTA	No subgoup	26	31	18:23^a^	114	108–120	All-inside, outside-in	16 (59%)^a^	FasT-Fix™ hybrid (Smith & Nephew)	Revision surgery + Arthro-CT	13.0
2000	Rockborn P et al. [26]	JBJSB	No subgoup	25^b^	31	5:26	162^b^	132–228	Open repair	0	No All-Inside	Revision surgery	29.0
2007	Siebold R et al. [29]	Arthroscopy	No subgoup	30	95	Not specified	72	60–80	All-inside	75 (66%)^a^	Meniscus Arrow (Bionx Implants)	Revision surgery	28.0
2016	Solheim E et al. [31]	KSSTA	No subgoup	33^b^	82	Not specified	120^b^	84–144	All-inside	0	RapidLoc™ (Depuy-Mitek)	Revision surgery	48.0
2015	Steadman JR et al. [33]	AJSM	No subgoup	33	148	Not specified	Not specified	120 Minimum	Inside-out	Not specified	No All-Inside	Revision surgery	5.0
2004	Steenbrugge F et al. [34]	KSSTA	Inside-Out	33.5	45	9:11	84	66–102	Inside-out	4 (9%)	Biofix™ Arrow (Bioscience)	Revision surgery	0.0
			Biofix™ Arrow	37.5		9:16			All-inside				8.0
2014	Westermann RW et al. [41]	AJSM	No subgoup	23.6	235	Not specified	72	72–72	All-inside, inside-out, outside-in	235 (100%)	Not specified	Revision surgery	14.0

Repair devices are specified for all-inside technique. All numbers refer to time at follow-up unless stated differently

*ACL* anterior cruciate ligament, *ACLR* anterior cruciate ligament reconstruction, *AJSM* American Journal of Sports Medicine, *BMI* Body Mass Index, *CT* computed tomography, *JBJSA* Journal of Bone and Joint Surgery, American Volume, *JBJSB* Journal of Bone and Joint Surgery, British Volume, *KSSTA* Knee Surgery, Sports Traumatology, Arthroscopy, *MR* meniscus repair, *Ø* average value

^a^At baseline

^b^Median value

**Table 3:** Tear and suture characteristics of all included studies (*n* = 12)

Tear and Suture characteristics									
Year	Author	Journal	Subgroups	Length	Type	Zone	Laterality (medial%/lateral%)	Suture details (technique, number, absorbable/non-absorbable)	Time between meniscus injury to surgery (months)
2019	Billières J et al. [5]	KSSTA	No subgoup	Not specified	Horizontal	Not specified	21/79^a^	Vertical sutures, Absorbable	Not specified
2014	Bogunovic L et al. [6]	JBJSA	Isolated MR	Not specified	Not specified	RR, RW	62/38	Not specified	Not specified
2005	Lee GP et al. [14]	AJSM	No subgoup	Ø 20.6 mm (15–35 mm)	Vertical longitudinal	RR, RW	Not specified	Ø 2.51 arrows (1–6)	Not specified
2009	Logan M et al. [15]	AJSM	No subgoup	Not specified	Bucket-handle, radial, complex tears	Not specified	67/33	Vertical sutures, Ø 3.7 (1–12), absorbable	Ø 7 (0–45)
2006	Majewski M et al. [19]	AJSM	No subgoup	Not specified	Vertical longitudinal	Not specified	57/43	Not specified	Not specified
2015	Pujol N et al. [25]	KSSTA	No subgoup	Not specified	Vertical	RR, RW	61/39^a^	Ø 3 sutures (1–7)	Ø 114 ± 10
2000	Rockborn P et al. [26]	JBJSB	No subgoup	20–40 mm	Vertical longitudinal, bucket-handle	Not specified	55/45	Not specified	13.5 ± 26 weeks^b^
2007	Siebold R et al. [29]	Arthroscopy	No subgoup	10–25 mm	Not specified	RR, RW	Not specified	Ø 2 arrows (1–4)	Ø 3 (0–21)
2016	Solheim E et al. [31]	KSSTA	No subgoup	≥ 10 mm	Bucket-handle	RR, RW	Not specified	Absorbable	Not specified
2015	Steadman JR et al. [33]	AJSM	No subgoup	Not specified	Not specified	Not specified	Not specified	(2–5), absorbable	Not specified
2004	Steenbrugge F et al. [34]	KSSTA	Inside-Out	Not specified	Not specified	RR, RW, WW	85/15	Not specified	Not specified
			Biofix™ Arrow				92/8	Not specified	Not specified
2014	Westermann RW et al. [41]	AJSM	No subgoup	Ø 16.5 mm	Vertical longitudinal, bucket-handle, horizontal, oblique, radial, complex	RR, RW, WW	68/32	Not specified	Not specified

All numbers refer to time at follow-up unless stated differently

*ACL* anterior cruciate ligament, *ACLR* anterior cruciate ligament reconstruction, *AJSM* American Journal of Sports Medicine, *JBJSA* Journal of Bone and Joint Surgery, American Volume, *JBJSB* Journal of Bone and Joint Surgery, British Volume, *KSSTA* Knee Surgery, Sports Traumatology, Arthroscopy, *MR* meniscus repair, *RR* Red–Red, *RW* Red–White, *WW* White–White, *Ø* average value

^a^At baseline

^b^Median value

### Status of the ACL

A total of six studies [5, 6, 19, 26, 29, 31] reported meniscus repair outcome in ACL intact knees and three studies [14, 29, 41] provided information on failure of meniscus repair when performed with concomitant ACLR. A total of 260 patients with an intact ACL and 338 patients with concomitant ACLR were analyzed. The pooled study results showed no statistically significant difference (n.s.) between meniscus repair failure rates in ACL intact knees 28% (95% CI 0.118–0.442) and ACLR knees18.7% (95% CI 0.020–0.354).

### Laterality of meniscus repair

**Fig. 2:**
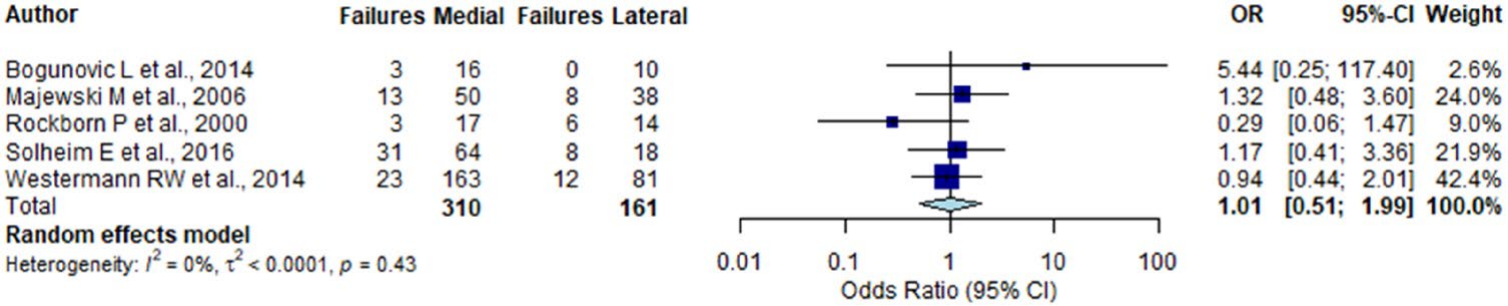
Random-effects model for the effect of failure rates between medial versus lateral meniscus repair (n.s.)

Five studies [6, 19, 26, 31, 41] stated detailed information on laterality of meniscus repair (medial or lateral) as well as the side of failure. This included a total of 310 medial and 161 lateral menisci. Pooled failure rate was 24.4% (95% CI 0.073–0.415) after medial repair and 19.5% (95% CI 0.007–0.383) after lateral meniscus repair, respectively. Subgroup analysis did not reveal a significant difference between failure rates of medial and lateral meniscus repair (n.s.). The overall pooled estimate did not reveal a significantly higher risk of failure for medial meniscus repair (OR 1.01; 95% CI 0.510–1.992, n.s.) (Fig. 2).

### Repair technique

All-inside repair was the most frequently used technique performed in a total of 464 cases in six studies [6, 14, 29, 31, 34, 41] with a pooled failure rate of 22.3% (95% CI 0.071–0.376). In four studies [15, 33, 34, 41], an inside-out technique was used accounting for 229 repairs with a pooled failure rate of 5.6% (95% CI 0.000–0.130). Two studies each reported on outside-in [19, 41] and open meniscus repair outcome [5, 26]. In 94 cases an outside-in repair was performed, and 44 tears underwent open meniscus repair representing a pooled failure rate of 23.2% (95% CI 0.000–0.493) and 23.0% (95% CI 0.000–1.091), respectively. Performing a subgroup analysis between all-inside and inside-out demonstrated a significant lower failure rate for inside-out meniscus repair (*p* = 0.009).

### Tear configuration

In five studies [14, 19, 25, 26, 41], a total of 377 vertical/longitudinal tears were treated with a pooled failure rate of 18.4% (95% CI 0.103–0.266). In three studies [26, 31, 41] failures of bucket-handle tear repair were reported, representing a total of 111 bucket-handle tears with a pooled failure rate of 29.9% (95% CI 0.000–0.867). Fifteen horizontal tears were treated in two studies [5, 41] representing a pooled failure rate of 16.2% (95% CI 0.000–0.506). A subgroup analysis did not reveal any significant difference between failure rates of vertical/longitudinal and bucket-handle tears (n.s.).

### Time of failure

**Fig. 3:**
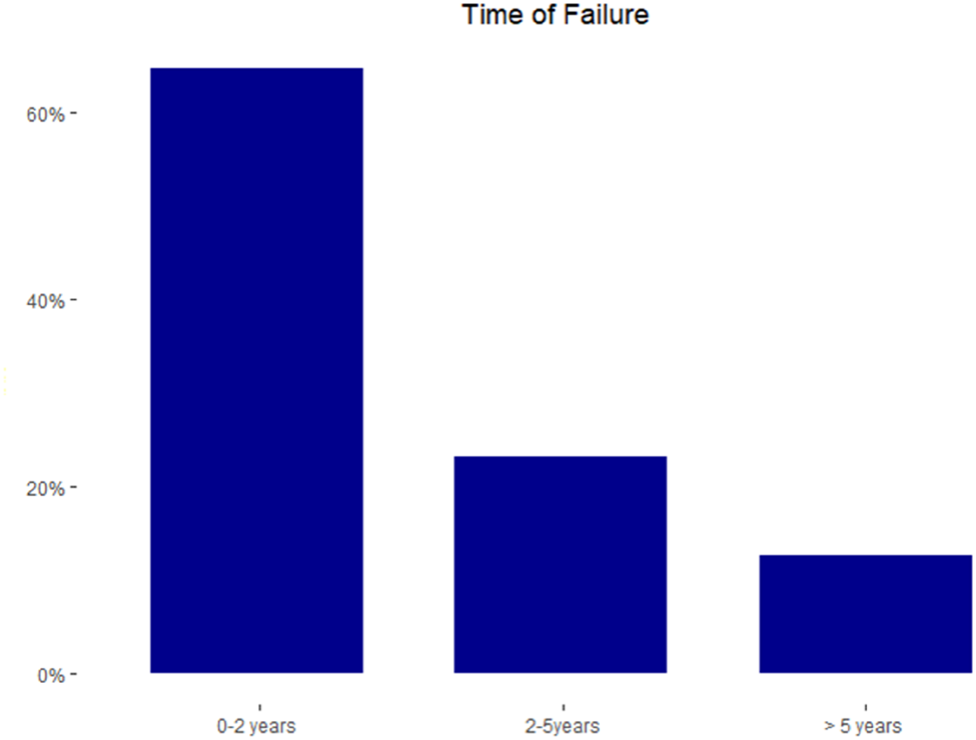
Histogram showing the time of failure

In total, 165 failures were reported in this analysis. Five studies [5, 25, 26, 31, 41] reported the time of failure accounting for 87 failures (53%). Of those, 64% (56/87) occurred within 2 years after meniscus repair. Between postoperative year 2 and 5, 23% (20/87) of failures were noted. After 5 years of index surgery, 13% (11/87) of failures were observed (Fig. 3). In summary, failures occurring after 2 years represented 36% (31/87) of all failures. Thirteen percent of failures were observed after the fifth postoperative year. The mean time of failure was reported in four other studies [6, 15, 19, 29] and ranged from 24 to 48 months.

### Age and postoperative mobilization

Repair outcome of patients younger than 40 was reported in three studies [15, 25, 33] with a pooled failure rate of 12.2% (95% CI 0.000–0.347). One study [33] reported failures in patients equal or older than 40 years with a failure rate of 5.3% (2/38). A total of 196 patients were mobilized partial weightbearing [5, 19, 29] and 59 patients full weightbearing [14, 25] with a pooled failure rate of 24.9% (95% CI 0.129–0.369) and 18.8% (95% CI 0.000–1.153), respectively. Failure rate for strict non weightbearing for 6 weeks was 5.4% (8/148) [33] and 11.5% (3/26) [6] for weightbearing as tolerated for 6 weeks. Range of motion was restricted to 90 degrees for at least 4 weeks (median 5 weeks) in 315 patients in five studies [5, 15, 25, 31, 33] with a pooled failure rate of 20.8% (95% CI 0.001–0.414). Range of motions up to 60 degrees for 6 weeks showed a failure rate of 23.9% (21/81) [19] and failure rate for gradual increase in range of motion [29], range of motion as tolerated [14] and immobilized knees [26] ranged between 28 and 29%.

## Discussion

The most important findings of the present meta-analysis were that meniscus repair yields in an overall failure rate of 19.1% at a mean follow-up of 86 months. More than one third (36%) of meniscus repairs fail after the second year and 13% after the fifth postoperative year.

In this study, failure rates were highly heterogenous and ranged from 4.4 [34] to 48% [31] with an overall failure rate of 19.1%. A previous meta-analysis [21] on the long-term outcome of meniscal repair reported a slightly higher overall failure rate (23.1%). The failure definition did not strongly differ among included studies (Table 2), concluding that heterogeneity on failure rates may be due to inherent variations in meniscus injuries, different operative techniques, and devices as well as patient´s characteristics and the postoperative treatment. The current study shows that meniscus repair frequently fails after the second postoperative year in overall 36%. Most studies defined failure as the need for repeat meniscus repair or subsequent partial meniscectomy without any stated cause of failure. Most of these studies did not specify whether revision surgery was performed in the location of previous tear or adjacent to the repair side on the same meniscus. This information would help to gain a better understanding on the re-tear pathomechanism.

There is a debate in the literature as to whether meniscus repair with concomitant ACLR yields superior results compared to isolated meniscus repair. Previous studies described a beneficial effect on meniscus healing when concomitant ACLR was performed [7, 38]. Furthermore, meniscus tears in combination with ACL rupture are related to an acute trauma and may undergo earlier surgery. On the other hand, there are reports that failed to show a better meniscus repair outcome in combination with ACLR [6, 36]. The current analysis demonstrated a trend (not statistically significant) towards a reduced risk of meniscus repair failure with concomitant ACLR. The slightly better meniscus repair outcome of meniscus injuries with concomitant ACL rupture may indicate a favorable healing potential of traumatic tears. However, it must be acknowledged that only three studies with a total of 338 included patients reported on the outcome of meniscal repair along with ACLR.

In current literature, studies [7, 23, 27] have shown lower failure rates of lateral meniscus repair compared to repair of the medial meniscus. The medial meniscus is more tightly fixed to the tibial plateau and the collateral ligament and therefore exposed to higher load. This may lead to increased failure rates on the medial side in comparison to the lateral side. In contrast to that, other studies failed to show any effect of laterality on meniscus repair outcome [3, 21, 41]. The current meta-analysis of studies with a minimum 5-year follow-up showed a trend towards a reduced failure rate of lateral meniscus repair. However, no significantly different failure rates of medial and lateral meniscal repair were observed. This study appears underpowered to demonstrate significant effects. Further studies with larger cohorts are therefore needed to evaluate the influence of laterality on meniscus repair outcome.

Various repair devices were introduced over the last years. This meta-analysis could demonstrate a large variation with respect to the selection of repair devices and associated failure rates. For example, two studies used the meniscus arrow (Bionx Implants) which has been associated with poor outcome [12, 14, 29]. Another study [31] reported the highest failure rate (48.1%) of all included studies using the RapidLoc™ (Depuy-Mitek) meniscal repair device. On the other hand, one study [6] demonstrated excellent survivorship with a failure rate of 12% using the FasT-Fix™ (Smith & Nephew Endoscopy) device. Unfortunately, the number of studies was too small to perform a subgroup analysis for different repair devices and not all studies in the current analysis have specified the repair device. However, a specification of repair devices would be essential in order compare the results among different studies. Similar pooled failure rates were demonstrated for all-inside, outside-in and open meniscus repair. Interestingly, pooled failure rate of inside-out repair in 229 cases was significantly lower with 5.6% compared to the all-inside technique. However, these results may be skewed as one study [33] using inside-out repair reported a very low failure rate (5.4%) and accounted for nearly two thirds of all analyzed inside-out repairs. Nonetheless, a recent meta-analysis found similar results after the comparison of all-inside versus inside-out repair with concomitant ACLR revealing a significant lower failure rate for inside-out repair (16% versus 10%) at a follow-up of 2 years [40]. However, the authors did not specifically evaluate different repair devices in the all-inside repair group. Due to limited number of studies and the great variation of cohort sizes among studies, superior outcome of inside-out repair has to be interpreted with caution. More evidence is warranted to determine whether success of meniscus repair depends on the selected repair method and repair device.

Vertical/longitudinal tears were the most frequently observed tears in the current meta-analysis. In comparison to bucket-handle tears, no significantly different failure rates were detected. It has been shown that horizontal tears often face suture failure caused by shear stresses [32], extension of tears into the non-vascularized zone with substantial degenerative components, reducing the chances of healing [11]. The current analysis only included fifteen horizonal tears in two study [5, 41] and therefore no subgroup analysis for horizontal tears was performed. Due to limited data, the repair outcome of horizontal tears was not evaluated. Moreover, the effect of patients’ age on meniscal repair outcomes needs to be investigated in further studies since available data was limited and the number of included studies was low.

Postoperative rehabilitation protocols have been advocated ranging from restricted rehabilitation regimes with no weightbearing and immobilization of the knee to accelerated approaches with full weightbearing and free range of motion. In this analysis, most studies proposed an accelerated postoperative mobilization with partial or full weightbearing and restriction of motion up to 90 degrees. Failure rates of different weightbearing regimes as well as the results of different motion restrictions were overall comparable. Based on the current literature no recommendation for specific rehabilitation protocol can be made.

One major limitation of this study is the variation in meniscus tear characteristics, repair methods and patients’ characteristics among included studies as well as the low numbers of long-term studies on meniscus repair outcome in the literature. There was only limited information available in terms of tear characteristics (zone of meniscus tear, chronicity, acute/degenerative), suture characteristics (number of sutures, absorbable/non-absorbable, suture technique) and additional patients characteristics (BMI, smoker status, level of activity). Additionally, the influence of age on the outcome of meniscus sutures is poorly described. Even though authors were contacted for additional data, there was a lack of detailed information for subgroup analyses, in particular for the above-mentioned patient-related and technique-related factors. Most of the studies did not specify the etiology (traumatic/degenerative) of the meniscus tear. However, the mean age of patients was under thirty and the current analysis only included fifteen horizontal tears which are most of the time degenerative. Another limitation is the retrospective study design of included studies. Due to these limitations, the results need to be interpreted with caution. However, while previous meta-analysis either included older studies or focused on the comparison between different repair techniques [21, 24], this meta-analysis concentrates on recently published studies including newer generation all-inside repair devices and reports the repair outcome of different tear configurations. In addition, no larger meta-analysis has been published before in the literature.

The findings of this study may help clinicians to educate patients about the expected results of meniscus sutures. Despite the given technical advantages of all-inside repair devices, this meta-analysis cannot demonstrate superior outcomes compared to inside-out or outside-in repair at 5 years. No recommendation for a specific repair device can be made. Furthermore, there is no gold standard in the postoperative rehabilitation as different weightbearing regimes as well as different motion restrictions regimes yielded in comparable outcomes.

## Conclusion

The current meta-analysis revealed an overall meniscus repair failure rate of 19.1% in studies with a minimum follow-up of 5 years. Thirty-six percent of meniscus repair failures occur after the second postoperative year. A trend towards better meniscus repair outcome when performed in combination with ACL reconstruction was observed. Furthermore, meniscus repair on the lateral meniscus tends to have a better healing response compared to the medial meniscus. A significantly better meniscus repair outcome could be demonstrated for the inside-out repair technique compared to all-inside repair. A subgroup analysis on failure rates of vertical/longitudinal and bucket-handle tears did not reveal any significant differences in outcome. The cause of failure is poorly documented, and it remains unclear whether failure of the meniscus repair itself or additional adjacent tears lead to revision surgery.

## Abbreviations

ACL: Anterior cruciate ligament
ACLR: Anterior cruciate ligament reconstruction
CI: Confidence intervals
IR: Incidence rate
MINORS: Methodological index for non-randomized studies
OR: Odds ratio
PRISMAL: Preferred reporting items for systematic reviews and meta-analyses
REM: Random-effect models
REML: Restricted maximum likelihood method
